# Graph Regularized Deep Sparse Representation for Unsupervised Anomaly Detection

**DOI:** 10.1155/2021/4026132

**Published:** 2021-11-03

**Authors:** Shicheng Li, Shumin Lai, Yan Jiang, Wenle Wang, Yugen Yi

**Affiliations:** School of Software, Jiangxi Normal University, Nanchang 330022, China

## Abstract

Anomaly detection (AD) aims to distinguish the data points that are inconsistent with the overall pattern of the data. Recently, unsupervised anomaly detection methods have aroused huge attention. Among these methods, feature representation (FR) plays an important role, which can directly affect the performance of anomaly detection. Sparse representation (SR) can be regarded as one of matrix factorization (MF) methods, which is a powerful tool for FR. However, there are some limitations in the original SR. On the one hand, it just learns the shallow feature representations, which leads to the poor performance for anomaly detection. On the other hand, the local geometry structure information of data is ignored. To address these shortcomings, a graph regularized deep sparse representation (GRDSR) approach is proposed for unsupervised anomaly detection in this work. In GRDSR, a deep representation framework is first designed by extending the single layer MF to a multilayer MF for extracting hierarchical structure from the original data. Next, a graph regularization term is introduced to capture the intrinsic local geometric structure information of the original data during the process of FR, making the deep features preserve the neighborhood relationship well. Then, a L1-norm-based sparsity constraint is added to enhance the discriminant ability of the deep features. Finally, a reconstruction error is applied to distinguish anomalies. In order to demonstrate the effectiveness of the proposed approach, we conduct extensive experiments on ten datasets. Compared with the state-of-the-art methods, the proposed approach can achieve the best performance.

## 1. Introduction

Anomaly detection (AD) aims at finding the part of data that do not conform with the expected behavior [1]. These data are usually called outliers, anomalies, and so on. The anomalies sometimes naturally represent the abnormal events, e.g., damage to the sensors, cyberattack, and black swan events in the financial sector. Therefore, a series of AD-based methods have been proposed to remove these outliers from the original data and applied in many application fields such as fraud detection, wireless sensor networks, medical diagnosis, and so on [2, 3].

AD-based methods can be roughly divided into the following three categories: supervised anomaly detection (SAD), semisupervised anomaly detection (SSAD), and unsupervised anomaly detection (UAD). SAD-based methods, e.g., support vector machine (SVM) [4, 5], can be regarded as a one-class classification (OCC) problem under the unbalanced samples. SSAD-based methods, e.g., one-class random forest [6], often use partially labeled data to train the model. Since these approaches depend on the labeled data to train the model, the insufficient labeled data will limit their performance. However, the unlabeled data are often enough and easy to obtain, so some researchers proposed UAD-based methods, which utilize unlabeled data to build model and classify the anomaly data points. For instance, local outlier factor (LOF) [7] defines a metric to calculate outlier score for every data point directly.

In UAD-based methods, the data are often collected from the high-dimensional space, which leads to the high computational cost and storage space. In this case, the distance-based methods [8, 9] cannot perform efficiently. Although some accelerate techniques [10] have been proposed to deal with the aforementioned issue, they are still not suitable for handing the complex data. Furthermore, the “Distance Concentration” phenomenon, as well as called “Curse of Dimensionality” problem, is prone to occur in the complex data, which leads to the distances among data points tend to become almost the same [11]. Under this circumstance, it is very hard to use the deviation to distinguish abnormal values from the normal values. Besides, the high-dimensional data always have a lot of irrelevant noise data, which interfere with the detection of outliers [12]. In order to overcome these problems, some scholars proposed some clustering-based approaches for anomaly detection. In these methods, feature representation methods such as subspace learning are used to transform the original high-dimensional data into the low-dimensional feature space. Then, the clustering algorithms are performed on the new feature representation of the original data to discover outliers [13, 14]. Although these methods can achieve better detection results, their performances may be greatly affected by both the quality of feature representation methods and the stability of the clustering algorithms. To reduce the influence of clustering algorithms, reconstruction error-based methods have been proposed in which the error can be regarded as the outlier score for anomaly detection [15, 16].

Learning more useful feature representation from the original data for detecting outliers is very important and also attracts too much attention. Matrix factorization (MF) is a brilliant framework for FR, which has been widely used for anomaly detection such as principal component analysis (PCA) [17] and nonnegative matrix factorization (NMF) [18]. Compared with PCA, NMF has obtained a more meaningful feature representation due to the fact that the nonnegative constraints are added during the procedure of MF. NMF aims to decompose the original matrix into the inner product of a nonnegative basis matrix and a nonnegative coefficient matrix. Therefore, the original samples can be represented as the linear combination of the basis matrix's column vector and the combination coefficient is the corresponding row of the coefficient matrix. Due to the nonnegative constraints, the learned components can be linearly added to represent the original samples, which make NMF be widely used in anomaly detection [19–22]. Tong et al. [23] propose a nonnegative residual matrix factorization (NRMF) framework, which finds misbehavioral IP sources and abnormal users. Kannan et al. [24] employ NMF to search the outliers from the text data. In addition, Alshammari et al. [25] do the similar work on wireless sensor networks' data. However, since the abovementioned methods ignore the structural information of data, their performances will be affected. To overcome this problem, some variants' NMF methods have been proposed. For example, Cai et al. [26] introduce the manifold learning into original NMF and propose graph regularized NMF (GNMF). GNMF regularizes the original NMF formulation by using a Laplacian matrix and the structural information can be preserved well. Kuang et al. [27] propose symmetric NMF (SNMF), which can not only takes the structure information into considered but also obtains a low-rank result. Recently, Ahmed et al. [28] consider the neighborhood structure similarity information and propose neighborhood structure-assisted NMF (NS-NMF). NS-NMF uses minimum spanning tree (MST) to characterize the structural information, which shows good performance in anomaly detection.

Different from NMF-based methods, sparse representation (SR) [29] is another MF-based approach and has received growing attention in many applications, e.g., denoising [30, 31], classification [32, 33], and pattern recognition [34, 35]. In the field of anomaly detection, SR-based methods also show powerful performances. For example, Cong et al. [36] propose the sparse reconstruction cost (SRC) over the normal dictionary and apply it to detect abnormal events. Similar to some density-based anomaly detection methods, Xiao et al. [37] introduce sparsity measurement on the original NMF to detect anomalies in surveillance video. Based on low rank (LR) and SR, Xu et al. [38] propose an anomaly detection method for hyperspectral images. Different from [38], Ling et al. [39] impose the sum-to-one and nonnegativity constraints to get physically meaningful result. Pilastre et al. [40] propose a method based on SR and dictionary learning (DL) which can handle multivariate telemetry time series described by mixed continuous and discrete parameters.

Since the original SR-based methods only focus on the approximation representation of the original data and ignore the intrinsic structure of the data, it can hardly deal with the complex data well. In other words, the new feature representation loses the local geometric structure of the original high-dimensional data. Actually, a pair of adjacent data in a high-dimensional feature space should maintain the same relationship in a new feature space. To achieve this goal, Zheng et al. [41] introduce the manifold learning into SR and design graph regularized sparse coding (GRSC). GRSC uses the Laplace matrix to measure the features so that the features can preserve the local geometric structure. Previous studies [26–28, 42] have also shown that the geometric structure of the data can help to detect abnormal points.

In addition, original SR-based methods belong to shallow feature representation framework, which can only extract the shallow representation of the data. To remedy this limitation, He et al. [43] propose a deep sparse coding (DSC) method which extends a single layer sparse coding to a three-layer deep network architecture model. Moreover, in order to learn more discriminative feature representation, Sharma et al. [44] added a dense layer between two sparse layers. Tariyal et al. [45] and Singh et al. [46] propose a deep dictionary learning (DDL) framework for image classification and nonintrusive load monitoring. Cheng et al. [47] propose a deep sparse representation (DSR) method, which integers a two-layer convolutional neural network (CNN) for extracting the high-level features and a sparse representation classifier (SRC) for face recognition. In addition, deep neural network (DNN) approaches including AutoEncoder (AE) [48] and Generative Adversarial Net (GAN) [49] have also been used in anomaly detection, but these approaches are easy to fall into overfitting, and the results are hard to interpret.

**Figure 1:**
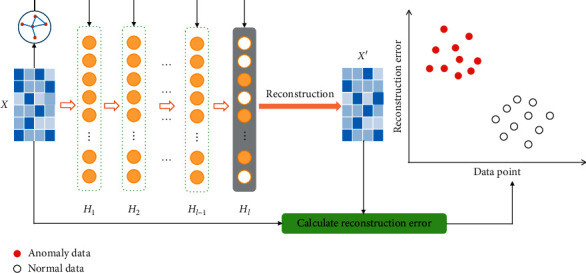
The illustration of the proposed GRDSR method.

Inspired by the works of [26, 41, 43], we propose a novel deep representation framework based on SR named as graph regularized deep sparse representation (GRDSR) for detecting anomaly data in the high-dimensional space, as shown in Figure 1. Similar to the residual block on the residual net [50], we introduce the graph regularization to the deep features on each layer to maintain the local geometric structure. Furthermore, the *L*_1_-norm is applied to learn the deep sparse representations to avoid overfitting. Unlike DNN-based anomaly detection methods, there are fewer parameters in our proposed approach. More importantly, the proposed approach is simpler and more straightforward, which can obtain interpretable results. The experiments are carried out on ten benchmark datasets and the experimental results verify the effectiveness of the proposed approach.

The main contributions of the proposed approach are given as below:

1. This paper employs a deep feature representation framework to detect anomalies. Different from the traditional single layer SR-based methods, the proposed framework performs deep representation on the coefficient matrix so that the obtained hierarchical deep feature representations are more discriminative.
2. Unlike the DNN-based methods, the proposed SR-based deep representation framework has a multilayer linear structure. Therefore, the extracted deep feature representations have stronger interpretability.
3. To make the deep feature representation preserve the intrinsic geometry of the original high-dimensional data, the graph regularization term is integrated into the deep feature representation framework by constructing a nearest neighbor graph to model the manifold structure. Besides, we impose a sparse constraint on the deep feature representations which makes the features be more sparsity and discriminative.

The rest of paper is organized as follows. In Section 2, there is a brief introduction of sparse coding and the graph regularization term.  Section 3 introduces the proposed method in detail. In Section 4, we conduct extensive experiments on public datasets to test the performance of the proposed method. And finally, we conclude our study in Section 5.

## 2. Related Works

In this section, we will make a brief introduction of sparse coding and the graph regularization.

### 2.1. Sparse Coding

Suppose that the *m*-dimensional data *X* has *n* samples (i.e., *X*=[*x*_1_,…, *x*_*n*_] ∈ *ℝ*^*m*×*n*^); spares' coding aims to find a dictionary matrix constructed by a set of basis vectors that capture high-level semantics from the original high-dimensional data. Let *W*=[*w*_1_,…, *w*_*k*_] ∈ *ℝ*^*m*×*k*^ be the over-complete dictionary matrix in which the *k* columns are called as atoms. *H*=[*h*_1_,…, *h*_*n*_] ∈ *ℝ*^*k*×*n*^ is the representation coefficient matrix. With the usage of dictionary *W*, the data sample *x*_*j*_ can be reconstructed as *x*_*j*_ ≈ ∑_*i*=1_^*k*^*w*_*i*_*H*_*ij*_. Therefore, *x*_*j*_ can be regarded as a sparse linear combination of new basis and *H*_*ij*_ is the combination coefficient.

Usually, spares' coding can be seen as an optimization problem and the objection function is defined as

(1) $$\begin{array}{c} O_{\text{SC}}=\underset{W,H}{\min}{\left\|X-WH\right\|}_{F}^{2}+\beta \overset{n}{\underset{i=1}{\sum }}f\left(h_{i}\right),\\ \text{s.t. }{\left\|w_{i}\right\|}^{2}\leq 1,\;i=1,\ldots ,k. \end{array}$$

where ‖•‖_*F*_ represents the Frobenius norm and *f*(•) is the function to measure the sparse. For convenience, *f*(•) can be chosen as the *L*_0_-norm, which counts the nonzero entries. Unfortunately, the optimization problem of equation (1) has been proven to be an NP-hard problem. Therefore, we use the *L*_1_-norm to replace the *L*_0_-norm so that it becomes a convex relaxation of the original problem and the objective function can be rewritten as

(2) $$\begin{array}{c} O_{\text{SC}}=\underset{W,H}{\min}{\left\|X-WH\right\|}_{F}^{2}+\beta \overset{n}{\underset{i=1}{\sum }}{\left\|h_{i}\right\|}_{1},\\ \text{s.t. }{\left\|w_{i}\right\|}^{2}\leq 1,\;i=1,\ldots ,k. \end{array}$$

Seen from equation (2), the objective function is convex in *W* or *H* only. To solve the factored matrices, one approach is to iteratively optimize the objective function, i.e., keep other variables fixed when updating one.

### 2.2. Graph Regularization

For given two data points *x*_*i*_ and *x*_*j*_, *h*_*i*_ and *h*_*j*_ are the corresponding feature representations with respect to the learned new basis. If *x*_*i*_ and *x*_*j*_ are close in the intrinsic geometry of the data distribution, then *h*_*i*_ and *h*_*j*_ are also close to each other, which is called locality assumption. To achieve locality assumption, the manifold structure of the high-dimensional data is introduced, which can be represented by a Laplacian matrix.

Firstly, we define a graph *G*=(*V*, *E*, *S*), where *V* is the set of nodes, *E* is the set of edges, and *S* is a weight matrix of *E*. Generally, some methods like *k*-NN firstly judge whether a pair of points is connected, and then, the weights on the edges are computed. There are many ways to compute the weight matrix. Here, three most commonly used methods are introduced as follows:

(1) 0-1 weighting:
  (3) $$\begin{array}{c} S_{ij}=\left\{\begin{array}{cc} 1, & \text{if nodes }i\text{ and }j\text{ are connected},\\ 0, & \text{others}. \end{array}\right. \end{array}$$
(2) Heat kernel weighting:
  (4) $$\begin{array}{c} S_{ij}=\left\{\begin{array}{cc} e^{-\left({\left\|x_{i}-x_{j}\right\|}^{2}/\sigma \right)}, & \text{if nodes }i\text{ and }j\text{ are connected},\\ 0, & \text{others,} \end{array}\right. \end{array}$$
where *σ* is the hyperparameter.
(3) Dot-product weighting:

(5) $$\begin{array}{c} S_{ij}=\left\{\begin{array}{cc} x_{i}^{T}x_{j}, & \text{if nodes }i\text{ and }j\text{ are connected},\\ 0, & \text{others}. \end{array}\right. \end{array}$$

Equation (5) can be equivalent to cosine similarity if *x* is normalized to 1. The weight matrix is also called the similarity matrix.

Then, the Euclidean distance is employed to measure the similarity of a pair of feature representation:

(6) $$\begin{array}{c} d\left(h_{i},h_{j}\right)={\left\|h_{i}-h_{j}\right\|}^{2}. \end{array}$$

Finally, the smoothness of the feature representation is measured by the similarity matrix, which is defined as follows:

(7) $$\begin{array}{c} R=\frac{1}{2}\sum_{i,j=1}^{n}{\left\|h_{i}-h_{j}\right\|}^{2}S_{ij}\\ =\text{Tr}\left(HDH^{T}\right)-\text{Tr}\left(HSH^{T}\right)\\ =\text{Tr}\left(HLH^{T}\right), \end{array}$$

where *D* is a diagonal matrix, *D*_*ii*_=∑_*j*_*S*_*ij*_, *L* is Laplacian matrix and *L* = *D* − *S*, and Tr(•) denotes the trace of a matrix.

## 3. The Proposed Method

In this section, the objection function of the proposed approach is introduced first. Next, an iteration scheme is proposed to solve the objection function. Then, a criterion for anomaly detection is provided. At last, convergence analysis of the proposed optimization algorithm is given.

### 3.1. The Objection of GRDSC

Firstly, similar to MF, we represent *X* into the inner product of matrixes *W* and *H*; therefore, the process can be represented by

(8) $$\begin{array}{c} O_{\text{MF}}=\underset{W,H}{\min}{\left\|X-WH\right\|}_{F}^{2}. \end{array}$$

Since the traditional MF method only contains a single layer structure, it just extracts the shallow features so that the learned basis may contain complex hierarchical information. To address this disadvantage, the deep representation framework is proposed. Different from the existing methods, we further decompose the learned basis to get a better higher-level feature representation from the original data. Moreover, the multilayer structure can also learn multiple hidden basis of the original data. The objective function of deep representation framework can be represented as

(9) $$\begin{array}{c} O_{\text{DeepMF}}=\underset{W_{l},H_{l}}{\min}{\left\|X-\Lambda_{l-1}W_{l}H_{l}\right\|}_{F}^{2}, \end{array}$$

where *l* is the layer number, Λ_*l*−1_=*W*_1_*W*_2_ ⋯ *W*_*l*−1_, Λ_0_=*I*_*m*_ is the identity matrix. and *W*_*i*_ and *H*_*i*_ are temporary variables generated in the calculation process.

Next, the aforementioned deep representation framework in equation (9) does not take the geometrical information of data into consideration, which may lead to the poor feature representation when the data have complex manifold structures. Therefore, in order to preserve the local geometric structure information, the graph regularize term is introduced to guide the feature representation, i.e., similar samples are grouped into the same cluster. The graph regularize term can be defined as follows:

(10) $$\begin{array}{c} O_{\text{Graph}}=\underset{H_{i}}{\min}\sum_{i=1}^{l}\text{Tr}\left(H_{i}LH_{i}^{T}\right). \end{array}$$

Then, in order to enhance the discriminant ability of the deep feature, a sparsity constraint of the deep feature representation is added, which can be defined as

(11) $$\begin{array}{c} O_{\text{sparsity}}=\underset{H_{i}}{\min}\sum_{i=1}^{n}{\left\|h_{i}^{l}\right\|}_{1}, \end{array}$$

where ‖•‖_1_ denote the *L*_1_-norm of vector.

At last, taking equations (9)–(11) into consideration, the objective function of the proposed method can be summarized as

(12) $$\begin{array}{c} O_{\text{GRDSC}}=\underset{W_{l},H_{l}}{\min}\left\{{\left\|X-\Lambda_{l-1}W_{l}H_{l}\right\|}_{F}^{2}+\alpha \overset{l}{\underset{i=1}{\sum }}\text{Tr}\left(H_{i}LH_{i}^{T}\right)+\beta \overset{n}{\underset{i=1}{\sum }}{\left\|h_{i}^{l}\right\|}_{1}\right\},\\ \text{s.t. }{\left\|w_{i}^{l}\right\|}^{2}\leq 1,\;\;i\in \left\{1,\ldots ,k_{l}\right\}, \end{array}$$

where *α* ≥ 0 and *β* ≥ 0 are two tradeoff parameters and *w*_*i*_^*l*^ and *h*_*i*_^*l*^ are the vectors of the final dictionary matrix *W*_*l*_ and the coefficient matrix *H*_*l*_, respectively.

### 3.2. The Optimization of GRDSC

Since the objective function in equation (12) is not convex in both *W*_*l*_ and *H*_*l*_, it is very hard to get the globally optimal solution. To deal with this problem, this paper proposes an iterative updating algorithm to achieve the local optimal solution. Similar to the expectation maximization algorithm, we update one variable and fix the rest variables, and all variables are alternately updated. Additionally, a layer-by-layer processing strategy is applied to simplify the algorithm flow. Since the last layer is different from others, we will deal with it separately.

#### 3.2.1. Update Rule for *i*th Layer (*i* < *l*)

Because of the objective function for each layer is similar, we just take the *i*th layer for instance. The optimal problem can be represented as

(13) $$\begin{array}{c} O_{\text{GRDSC}}^{i}=\underset{W_{i},H_{i}}{\min}\left\{{\left\|X-\Lambda_{i-1}W_{i}H_{i}\right\|}_{F}^{2}+\alpha \sum_{i=1}^{l-1}\text{Tr}\left(H_{i}LH_{i}^{T}\right)\right\}. \end{array}$$

First, using the correlation properties of the matrix, we rewrite the objective function as

(14) $$\begin{array}{c} J_{i}=\text{Tr}\left({\left(X-\Lambda_{i-1}W_{i}H_{i}\right)}^{T}\left(X-\Lambda_{i-1}W_{i}H_{i}\right)\right)+\alpha \text{Tr}\left(H_{i}LH_{i}^{T}\right)\\ =\text{Tr}\left(X^{T}X-2X^{T}\Lambda_{i-1}W_{i}H_{i}+H_{i}^{T}W_{i}^{T}\Lambda_{i-1}^{T}\Lambda_{i-1}W_{i}H_{i}\right)+\alpha \text{Tr}\left(H_{i}LH_{i}^{T}\right). \end{array}$$

Then, the Lagrange function is

(15) $$\begin{array}{c} ℒ=\text{Tr}\left(X^{T}X\right)-2\text{Tr}\left(X^{T}\Lambda_{i-1}W_{i}H_{i}\right)+\text{Tr}\left(H_{i}^{T}W_{i}^{T}\Lambda_{i-1}^{T}\Lambda_{i-1}W_{i}H_{i}\right)+\alpha \text{Tr}\left(H_{i}LH_{i}^{T}\right). \end{array}$$

Taking partial derivation of ℒ with respect to *W*_*l*−_ and *H*_*l*_, respectively, we have

(16) $$\begin{array}{c} \frac{\partial ℒ}{\partial W_{i}}=-2\Lambda_{i-1}^{T}XH_{i}^{T}+2\Lambda_{i-1}^{T}\Lambda_{i-1}W_{i}H_{i}H_{i}^{T},\\ \frac{\partial ℒ}{\partial H_{i}}=-2W_{i}^{T}\Lambda_{i-1}^{T}X+2W_{i}^{T}\Lambda_{i-1}^{T}\Lambda_{i-1}W_{i}H_{i}+2\alpha H_{i}L. \end{array}$$

Setting (∂ℒ/∂*W*_*i*_)=0 and (∂ℒ/∂*H*_*i*_)=0, we have

(17) $$\begin{array}{c} W_{i}^{*}={\left(\Lambda_{i-1}^{T}\Lambda_{i-1}\right)}^{+}\Lambda_{i-1}^{T}XH_{i}^{T}{\left(H_{i}H_{i}^{T}\right)}^{+}, \end{array}$$

(18) $$\begin{array}{c} W_{i}^{T}\Lambda_{i-1}^{T}\Lambda_{i-1}W_{i}H_{i}+\alpha H_{i}L=W_{i}^{T}\Lambda_{i-1}^{T}X. \end{array}$$

where (•)^+^ denotes the pseudoinverse. See from equation (18), it is the Sylvester equation, and the optimal solution of *H*_*l*_ can be solved by Matlab function lyap.

#### 3.2.2. Update Rule for *l*th Layer

The optimization in the last layer is different from other layers because of the sparse regularization term. The objective function of the last layer can be represented as follows:

(19) $$\begin{array}{c} O_{\text{GRDSC}}^{l}=\underset{W_{l},H_{l}}{\min}\left\{{\left\|H_{l-1}-W_{l}H_{l}\right\|}_{F}^{2}+\alpha \text{Tr}\left(H_{l}LH_{l}^{T}\right)+\beta \overset{n}{\underset{i=1}{\sum }}{\left\|h_{i}^{l}\right\|}_{1}\right\},\\ \text{s.t. }{\left\|w_{i}^{l}\right\|}^{2}\leq 1,\;i\in \left\{1,\ldots ,k_{l}\right\}. \end{array}$$

Under a layer-by-layer processing strategy, the update of the previous layer has been completed and *H*_*l*__− 1_ has already been obtained. Next, we will discuss how to solve *W*_*l*_ and *H*_*l*_.

Computation of *W*_*l*_: when *H*_*l*_ is fixed, the dictionary *W*_*l*_ needs to be learned at first, and the problem of *W*_*l*_ can be described as

(20) $$\begin{array}{c} \phi \left(W_{l}\right)=\min{\left\|H_{l-1}-W_{l}H_{l}\right\|}_{F}^{2},\\ \text{s.t. }{\left\|w_{i}^{l}\right\|}^{2}\leq 1,\;i\in \left\{1,\ldots ,k_{l}\right\}. \end{array}$$

Suppose that Γ={*γ*_1_,…, *γ*_*k*_*l*__} is the Lagrange multiplier corresponding to ‖*w*_*i*_‖^2^ − 1 ≤ 0. Then, we can get the Lagrange dual function as

(21) $$\begin{array}{c} g\left(\Gamma \right)=\inf_{W}L_{w}\left(W_{l},\Gamma \right)\\ =\inf_{w}\left({\left\|H_{l-1}-W_{l}H_{l}\right\|}_{F}^{2}+\sum_{i=1}^{k_{l}}\gamma_{i}\left({\left\|w_{i}\right\|}^{2}-c\right)\right). \end{array}$$

And, *L*_*W*_(*W*, Γ) can be written as

(22) $$\begin{array}{c} L_{W}\left(W_{l},\Gamma \right)={\left\|H_{l-1}-W_{l}H_{l}\right\|}_{F}^{2}+\text{Tr}\left(W^{T}WA\right)-\text{Tr}\left(A\right)\\ =\text{Tr}\left(H_{l-1}^{T}H_{l-1}-2W_{l}^{T}H_{l-1}H_{l}^{T}+H_{l}^{T}W_{l}^{T}W_{l}H_{l}\right)\\ +\text{Tr}\left(W^{T}WA\right)-\text{Tr}\left(A\right), \end{array}$$

where *A* is a diagonal matrix and *A*_*ii*_=*γ*_*i*_. Then, partial derivation of equation (22) with respect to *W*_*l*_ is

(23) $$\begin{array}{c} \frac{\partial L_{W}\left(W_{l},\Gamma \right)}{\partial W_{l}}=W_{l}H_{l}H_{l}^{T}-H_{l-1}H_{l}^{T}+W_{l}A. \end{array}$$

Let equation (23) be equal to zero, and we have

(24) $$\begin{array}{c} W_{l}^{*}=H_{l-1}H_{l}^{T}{\left(H_{l}H_{l}^{T}+A\right)}^{+}. \end{array}$$

Then, substituting equation (24) into equation (22), we have

(25) $$\begin{array}{c} g\left(\Gamma \right)=\text{Tr}\left(H_{l-1}^{T}H_{l-1}\right)-\text{Tr}\left(H_{l-1}H_{l}^{T}{\left(H_{l}H_{l}^{T}+A\right)}^{+}H_{l}H_{l-1}^{T}\right)-\text{Tr}\left(A\right). \end{array}$$

From equation (25), we can get the following Lagrange dual function:

(26) $$\begin{array}{c} \underset{A}{\min}=\text{Tr}\left(H_{l-1}H_{l}^{T}{\left(H_{l}H_{l}^{T}+A\right)}^{+}H_{l}H_{l-1}^{T}\right)+\text{Tr}\left(A\right),\\ \text{s.t. }\gamma_{i}\geq 0,\;i\in \left\{1,\ldots ,k_{l}\right\}. \end{array}$$

It is obvious that the aforementioned problem can be solved by employing conjugate gradient or Newton's method. Supposing that *A*^*∗*^ is the optimal solution and the optimal of *W*_*l*_^*∗*^ can be computed as

(27) $$\begin{array}{c} W_{l}^{*}=H_{l-1}H_{l}^{T}{\left(H_{l}H_{l}^{T}+A^{*}\right)}^{+}. \end{array}$$

Computation of *H*_*l*_: after the dictionary *W*_*l*_ is fixed, the optimal problem of *H*_*l*_ can be defined as follows:

(28) $$\begin{array}{c} \underset{H_{l}}{\min}\left\{{\left\|H_{l-1}-W_{l}H_{l}\right\|}_{F}^{2}+\alpha \text{Tr}\left(H_{l}LH_{l}^{T}\right)+\beta \sum_{i=1}^{n}{\left\|h_{i}^{l}\right\|}_{1}\right\}. \end{array}$$

We can see that equation (28) is convex, but is nondifferentiable because of the *l*_1_-regularization. Following the work of [41], we will adopt an optimization method based on coordinate descent to solve this issue.

The vector *h*_*i*_^*l*^ should be updated individually and other vectors are fixed unchanged. So, we rewrite equation (26) as

(29) $$\begin{array}{c} \min\left\{\sum_{i=1}^{n}{\left\|h_{i}^{l-1}-W_{l}h_{i}^{l}\right\|}^{2}+\alpha \sum_{i,j=1}^{n}L_{ij}{\left(h_{i}^{l}\right)}^{T}h_{j}^{l}+\beta \sum_{i=1}^{n}{\left\|h_{i}^{l}\right\|}_{1}\right\}. \end{array}$$

And, the optimization problem about *h*_*i*_^*l*^ is

(30) $$\begin{array}{c} \underset{h_{i}^{l}}{\min}f\left(h_{i}^{l}\right)={\left\|h_{i}^{l-1}-W_{l}h_{i}^{l}\right\|}^{2}+\alpha L_{ii}{\left(h_{i}^{l}\right)}^{T}h_{i}^{l}+2\alpha {\left(h_{i}^{l}\right)}^{T}\left(\sum_{i\neq j}L_{ij}h_{j}^{l}\right)+\beta \sum_{j=1}^{n}\left|{\left(h_{i}^{l}\right)}^{j}\right|. \end{array}$$

where (*h*_*i*_^*l*^)^*j*^ is the *j*th coefficient of *h*_*i*_^*l*^.

We use subgradients of *f*(*h*_*i*_^*l*^) to deal with the nondifferentiable points; therefore, equation (30) can be rewritten as

(31) $$\begin{array}{c} f\left(h_{i}^{l}\right)=v\left(h_{i}^{l}\right)+\beta \sum_{j=1}^{n}\left|{\left(h_{i}^{l}\right)}^{j}\right|, \end{array}$$

where *v*(*h*_*i*_^*l*^)=‖*h*_*i*_^*l*−1^ − *W*_*l*_*h*_*i*_^*l*^‖^2^+*αL*_*ii*_(*h*_*i*_^*l*^)^*T*^*h*_*i*_^*l*^+2*α*(∑_*i*≠*j*_*L*_*ij*_*h*_*i*_^*l*^). This problem of equation (31) can be solved by feature-sign search algorithm proposed in [51].

**Figure 2:**
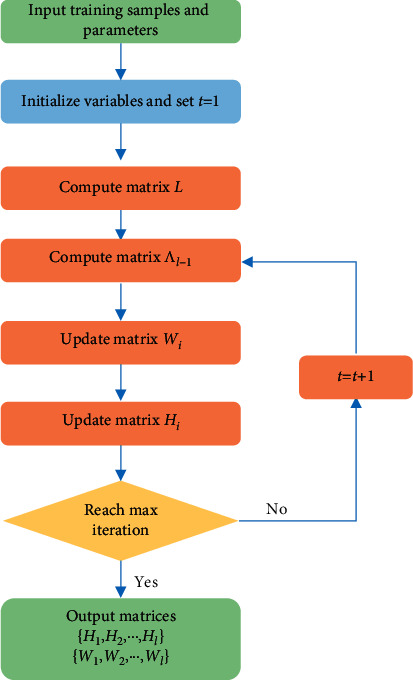
A flowchart of the proposed optimization algorithm.

The optimization algorithm of GRDSC is summarized in Algorithm 1, and algorithm flowchart is shown in Figure 2.

### 3.3. Anomaly Detection

In this section, we will give the description of the anomaly detection using the proposed GRDSC approach. Similar to SR-based methods for anomaly detection, the reconstruction error is employed to distinguish anomalies. Because the anomalies and normal data belong to different distribution and the number of anomalies is much less than normal data, the model is easily learned from classes with a large number of samples and ignores classes with a small number of samples. In other words, the reconstruction quality of anomalies is poor that has a higher anomaly score. Once the optimal *W*_*l*_^*∗*^, *H*_*l*_^*∗*^, and Λ_*l*_^*∗*^ are obtained, the reconstruction error between the original data and the reconstruction data is measured as follows:

(32) $$\begin{array}{c} \text{RE}\left(X,X^{\prime }\right)={\left\|X-X^{\prime }\right\|}_{F}^{2}={\left\|X-\Lambda_{l-1}^{*}W_{l}^{*}H_{l}^{*}\right\|}_{F}^{2}, \end{array}$$

where *X*′ denotes as the reconstruction data.

Furthermore, for every single sample, the reconstruction error can be computed as

(33) $$\begin{array}{c} O_{j}={\left\|x_{j}-x_{j}^{\prime }\right\|}_{F}^{2}={\left\|x_{j}-\sum_{i=1}^{k}{\left(\Lambda_{l-1}^{*}W_{l}^{*}\right)}_{i}{\left(H_{l}^{*}\right)}_{ij}\right\|}_{F}^{2}, \end{array}$$

where (*W*_*l*_^*∗*^)_*i*_ is the *i*th column in the *W*_*l*_^*∗*^. Then, we rank the score set {*O*_*j*_,  *j*=1,…, *n*} in descending order and those samples with high anomaly scores are marked as anomalies. The anomaly detection process is summarized in Algorithm 2.

### 3.4. Convergence Analysis

We will discuss the convergence of the proposed algorithm in this section. The optimization process can be divided into two subproblems as formulated in equations (13) and (19). Then, each subproblem can be divided into two subproblems. Thus, four subproblems can be solved iteratively. Let *ϕ*(*W*_*i*_, *H*_*i*_, *W*_*l*_, *H*_*l*_) be the objective function value of GRDSR, and we have the following theorem.

**Theorem 1 .**

The objection function value is nonincremental if Algorithm 1 is used to solve *ϕ*(*W*_*i*_, *H*_*i*_, *W*_*l*_, *H*_*l*_).

**Proof**

Let *ϕ*(*W*_*i*_^*t*^, *H*_*i*_^*t*^, *W*_*l*_^*t*^, *H*_*l*_^*t*^) denote the value of objective function in the *t*th iteration. We first can solve the subproblem min_*W*_*i*__*ϕ*(*W*_*i*_, *H*_*i*_^*t*^, *W*_*l*_^*t*^, *H*_*l*_^*t*^) when fix *H*_*i*_^*t*^, *W*_*l*_^*t*^, and *H*_*l*_^*t*^. The optimal solution *W*_*i*_^*t*+1^ in the *t* + 1th iteration can be obtained via equation (17). Since the subproblem is convex, we can obtain

(34) $$\begin{array}{c} \phi \left(W_{i}^{t+1},H_{i}^{t},W_{l}^{t},H_{l}^{t}\right)\leq \phi \left(W_{i}^{t},H_{i}^{t},W_{l}^{t},H_{l}^{t}\right). \end{array}$$

Next, by fixing *W*_*i*_^*t*^, *W*_*l*_^*t*^, and *H*_*l*_^*t*^, we can solve the subproblem min_*H*_*i*__*ϕ*(*W*_*i*_^*t*^, *H*_*i*_, *W*_*l*_^*t*^, *H*_*l*_^*t*^). The optimal value of *H*_*i*_^*t*+1^ can be obtain by solving equation (18). Since this subproblem is a convex problem, then we have

(35) $$\begin{array}{c} \phi \left(W_{i}^{t},H_{i}^{t+1},W_{l}^{t},H_{l}^{t}\right)\leq \phi \left(W_{i}^{t},H_{i}^{t},W_{l}^{t},H_{l}^{t}\right). \end{array}$$

Then, we fix *W*_*i*_^*t*^, *H*_*i*_^*t*^, and *H*_*l*_^*t*^ to solve the subproblem min_*W*_*l*__*ϕ*(*W*_*i*_^*t*^, *H*_*i*_^*t*^, *W*_*l*_, *H*_*l*_^*t*^). We can obtain the close solution by equation (24) according to literature [41], so this subproblem is convex, and we can obtain

(36) $$\begin{array}{c} \phi \left(W_{i}^{t},H_{i}^{t},W_{l}^{t+1},H_{l}^{t}\right)\leq \phi \left(W_{i}^{t},H_{i}^{t},W_{l}^{t},H_{l}^{t}\right). \end{array}$$

Similarly, we solve the subproblem min_*H*_*l*__*ϕ*(*W*_*i*_^*t*^, *H*_*i*_^*t*^, *W*_*l*_^*t*^, *H*_*l*_^*t*^) as depicted in equation (28) by fixing *W*_*i*_^*t*^, *H*_*i*_^*t*^, and *W*_*l*_^*t*^. Then, we can obtain

(37) $$\begin{array}{c} \phi \left(W_{i}^{t},H_{i}^{t},W_{l}^{t},H_{l}^{t+1}\right)\leq \phi \left(W_{i}^{t},H_{i}^{t},W_{l}^{t},H_{l}^{t}\right). \end{array}$$

Combining equations (34)–(37), we can obtain

(38) $$\begin{array}{c} \phi \left(W_{i}^{t+1},H_{i}^{t+1},W_{l}^{t+1},H_{l}^{t+1}\right)\leq \phi \left(W_{i}^{t},H_{i}^{t},W_{l}^{t},H_{l}^{t}\right). \end{array}$$

Therefore, Theorem 1 is proved.

At last, because the Frobenius norm, *L*_1_-norm, and trace are nonnegative, the objective function value in equation (12) is nonnegative, which has a low bound. In accordance with Cauchy convergence criterion and Theorem 1, the optimization algorithm for GRDSR is convergence.

## 4. Experiment Results and Analysis

To evaluate the performance of the proposed method, we conduct extensive experiments on real-world anomaly detection datasets and compare it with the state-of-art methods. The results show that the proposed method achieves better performance on most of the evaluated datasets.

### 4.1. Datasets' Descriptions

The datasets are chosen randomly from the study of Campose et al. [52]. Follow the work of [28], the missing values are removed and categorical variables are converted into numerical format. Besides, all of the data are normalized. The detail descriptions of the datasets are given as below, and a brief summary of the datasets is also shown in Table 1.

Annthyroid is a medical dataset about hypothyroidism, which contains three classes as normal (not hypothyroid), hyperfunction, and subnormal functioning. For anomaly detection, we treat hyperfunction and subnormal classes as abnormal.

Spambase is a dataset representing emails categorized as spam (outliers) or nonspam. The spam emails come from postmaster and individuals who had filed spam.

Wisconsin Prognostic Breast Cancer (WPBC) is collected from patients seen by Dr. Wolberg since 1984. Each sample represents follow-up data for one breast cancer case. The class *R* (recur) is marked as anomaly and the class *N* (nonrecur) is marked as normal.

Cardiotocography is a medical dataset which consists of measurements of fetal heart rate (FHR) and uterine contraction (UC) features on cardiotocograms. It is classified into normal, suspect, and pathologic by experts. For anomaly detection, the suspect class is discarded.

Ionosphere contains signals' data from good radars and bad radars in ionosphere where the ‘bad' class is treated as anomaly and ‘good' class is regarded as normal.

WBC records the measurements for breast cancer cases including benign and malignant two classes, where the malignant is considered as anomaly.

Arrhythmia is a multiclass classification dataset which contains 15 type of cardiac arrhythmia. The healthy people are treated as normal data and patients are marked as anomaly.

Pen digits collected 250 samples from 44 writers which are classified into 10 classes (0 … 9). In the experiment, Class 4 is defined as anomaly.

Stamps contain genuine stamps and forged stamps. The genuine stamps are using ink to print and treated as normal data. The forged data are photocopied or scanned and treated as anomaly.

Heart is an image dataset which describes diagnosing of cardiac Single Proton Emission Computed Tomography (SPECT). The original data are downsampled and affected patients are considered anomaly.

### 4.2. Score Metrics

As mentioned before, we compute the reconstruction error for each sample and obtain an anomaly score set {*O*_*j*_, *j*=1,…, *n*}. The higher score the observations associated with, the higher probability it be flagged as anomalies. However, the cut-off threshold is hard to selection. A common and widely used approach in practice is to select the top *N* instances and mark these as potential anomalies. In this paper, we follow this approach to mark the top *N* samples as anomalies and treat the rest as normal instances. For better evaluating the performance of the proposed method, we set *N* as the number of total anomalies in corresponding datasets.

Furthermore, the metric called precision at *N*(*P*@*N*) [52] is adopted to evaluate the performance of all of the methods. *P*@*N* is a straightforward metric and defined as the proportion of true outliers in the detected values which can be flagged as anomalies. Considering a dataset DB with *n* instances, *O* ⊂ DB is the anomaly set and *I*⊆DB is the normal data set, DB=*O* ∪ *I*. *P*@*N* is defined as

(39) $$\begin{array}{c} P@N=\frac{\#\left\{o\in O|\text{rank}\left(o\right)\leq N\right\}}{N}, \end{array}$$

where *N*=|*O*| is the number of anomaly samples.

### 4.3. Visualization Results and Analysis

**Figure 3:**
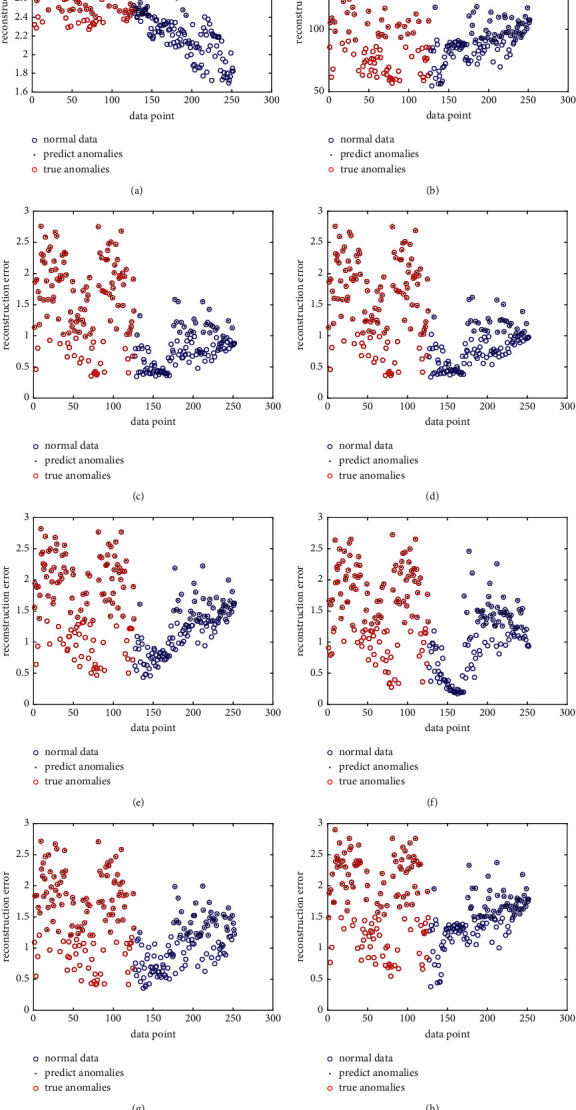
Reconstruction error for MF-based methods on ionosphere dataset. (a) GRDSR, (b) SNMF, (c) GraphSC, (d) SR, (e) Offline NS-NMF, (f) Online NS-NMF, (g) NMF, and (h) GNMF.

To more directly show the results of all of the detection processes, we plot the reconstruction error. Considering the proposed GRDSR method is based on MF in essence, we also choose the MF-based approaches for comparison. The selected comparison approaches include graph regularized sparse coding (GraphSC) [41], sparse representation (SR) [33], offline neighborhood structure-assisted NMF (Offline NS-NMF) [28], Online NS-NMF [28], graph regularized NMF (GNMF) [26], and symmetric NMF (SNMF) [27]. Besides, the ionosphere dataset is selected as representative for simplify. Considering the visualization results of compared algorithms may be affected by sample imbalance, we randomly select normal samples to balance the abnormal samples. The results are shown in Figure 3.

Form Figure 3, we can easily see that the reconstruction error calculated by the proposed GRDSR method is naturally divided into two parts, which means that the anomalies have bigger reconstruction error. Meanwhile, most of the normal data have smaller reconstruction error and distributed at the bottom right of the figure. In contrast to GRDSR, other MF-based methods have more difficulties to distinguish anomalies and normal data by the reconstruction error.

### 4.4. Comparison with the State-of-the-Art Methods

To further explore the performance of the proposed method, in this section, we first test the MF-based methods on all of the datasets. Since initialization is very important for MF-based methods, we fix the initialize method for all of the approaches. For GraphSC, SR, and GRDSR, we turn graph regularization parameter *α* and sparse regularization parameter *β* from a set {10^−3^, 10^−2^, 10^−1^, 1, 10, 10^2^, 10^3^} and report the best result. Following the work of [28], for all single-layer methods, the number of latent features or clusters *d* is set as 5. This is because changing *d* in the range of [5, 15] does not affect MF-based methods when *d* = 5 makes most of the methods perform well. For Offline NS-NMF, we set *α* = 0.8 and *γ* = 0.2; for Online NS-NMF, we set *α* = 0.8 and *z* = 20. In GNMF, we set the neighborhood graph construction parameter *k* in *k*NN as 5. Besides, we use 0-1 weighting as the weighting method. For SNMF, Gaussian similarity measure is utilized to construct the input similarity matrix.

Additionally, for fair comparison, the similarity matrix is constructed in our model identical to GraphSC and GNMF. In this experiment, the number of the hidden features for each layer, i.e., the layers' size, is set as [(*m*/2), (*m*/4)], where *m* means the dimension of the dataset. In practice, *m*/2 and *m*/4 are rounded to the nearest integer values. The settings for all of the MF-based methods are summarized in Table 2. The results are shown in Table 3. From Table 3, we can draw the following conclusions. Firstly, GNMF, Online NS-NMF, and Offline NS-NMF perform better than NMF and SNMF. Moreover, GraphSC performs better than SR. These results demonstrate that the graph regularization is helpful to preserve the intrinsic geometry during the process of feature representation. Secondly, GraphSC can achieve better performance than GNMF, which proves that the sparse representation with sparsity constraint can improve the discriminant ability of feature representation. Finally, compared with all MF-based methods, the proposed method either performs better or achieves the same best performance on all datasets except ionosphere dataset. This proves the effectiveness of the proposed GRDSR method under the deep framework based on SR for anomaly detection.

Then, in order to fully evaluate the performance of the proposed GRDSR method, we compare the proposed GRDSR method with other non-MF-based methods. Hence, 12 nearest neighborhood-based methods are chosen for comparison. These methods are *k*NN [8], kNN weight (kNNW) [53], local outlier factor (LOF) [7], outlier detection using indegree number (ODIN) [8], local distance-based outlier factor (LDOF) [9], connectivity-based outlier factor (COF) [54], local outlier probabilities (LoOP) [55], influenced outlierness (INFLO) [56], local density factor (LDF) [57], fast angle-based outlier detection (FastABOD) [58], and kernel density estimation outlier detection (KDEOD) [59]. Among them, kNN, ODIN, and kNNW can be seen as global methods. Another large category is derived from LOF, which can be seen as local methods. Besides, we also employ two DNN-based methods for comparison. They are autoencoder with an embedding regularizer (AER) [60] and deep autoencoding Gaussian mixture model (DAGMM) [61].

The number of the nearest neighbors (*k*) is required to be set in non-NMF-based methods. According to the guideline of [62], this paper tunes the values of *k* from 1 to 100 and the best value will be chosen. In our experiment, we only report the true positive detection number of all of the test methods. The results compared with non-NMF-based methods and DNN-based methods are shown in Table 4. The bolded entries mean the best performance in the corresponding datasets. Seen from Table 4, our proposed GRDSR method performs better in most cases except for the DANGMM and FABOD methods and achieves the best results on the Annthyroid, Pen digits, and Stamps datasets, respectively. In additional, the DANGMM method performs too much better than other methods on the Annthyroid dataset. Generally speaking, the proposed GRDSR method has made great progress than most of NMF-based methods and all non-NMF-based methods.

### 4.5. Parameter Sensitive Analysis

The proposed method has two trade off parameters, *α* and *β*, which are needed to be set at the beginning. In order to explore the settings of these parameters on each dataset, we conduct extensive experiments. As mentioned above, these parameters are set varied in the range of {10^−3^, 10^−2^, 10^−1^, 1, 10, 10^2^, 10^3^}, and we use a grid-search strategy to find the best parameter settings. The combinations of optimal parameters on different datasets are reported on Table 5. From this table, we can see that these parameters need to be set at a small value to reach a good performance in most cases. Compared with *β*, *α* often behaves smaller. This phenomenon shows that all datasets have a strong local structure.

**Figure 4:**
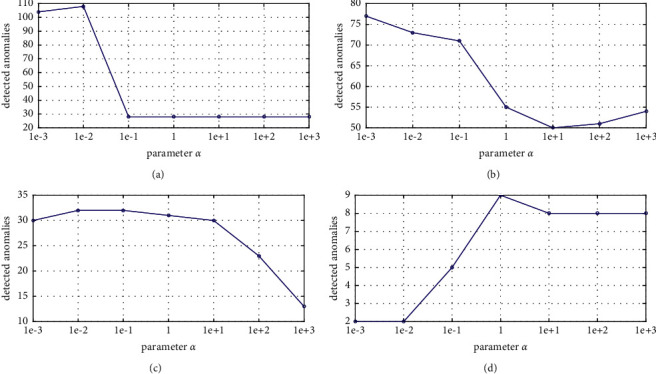
The performance of the GRDSR method with varied *α* on different datasets. (a) Ionosphere, (b) SpamBase, (c) Cardiotocography, and (d) WBC.

**Figure 5:**
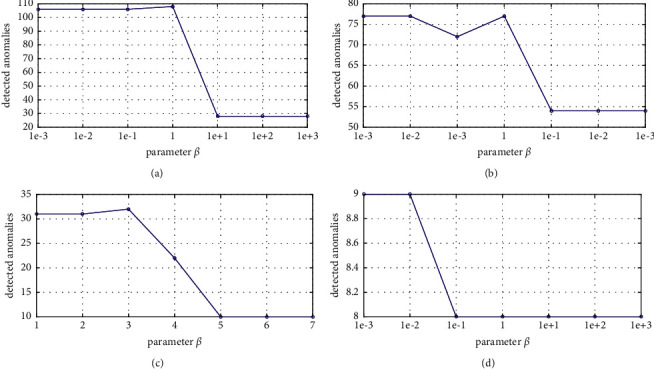
The performance of GRDSR method with varied *β* on different datasets. (a) Ionosphere, (b) SpamBase, (c) Cardiotocography, and (d) WBC.

In order to further visualize the influence of these two parameters, we randomly select four datasets. In order to make the visualization results more intuitively, we show one parameter and keep another fixed at the best. The results are reported on Figures 4 and 5. From Figure 4, it can be observed that, for Ionosphere, Cardiotocography, and SpamBase, the performances are first improved with the increase of the values of *α*. However, when the performances reached at the best, the performances begin to reduce or keep stable. However, for WBC, the trend is converse. This may be the characteristic of this datasets, that is to say, the sparsity is weaker than others so that the penalty factor needs to be set at a bigger value. From Figure 5, we can see that the trend seems to be identical for all datasets. The performance is stable when *β* is small. However, when *β* exceeds a certain value, performance will be decreased until it is stable again. The thresholds are different on different datasets.

### 4.6. Convergence Evaluation

**Figure 6:**
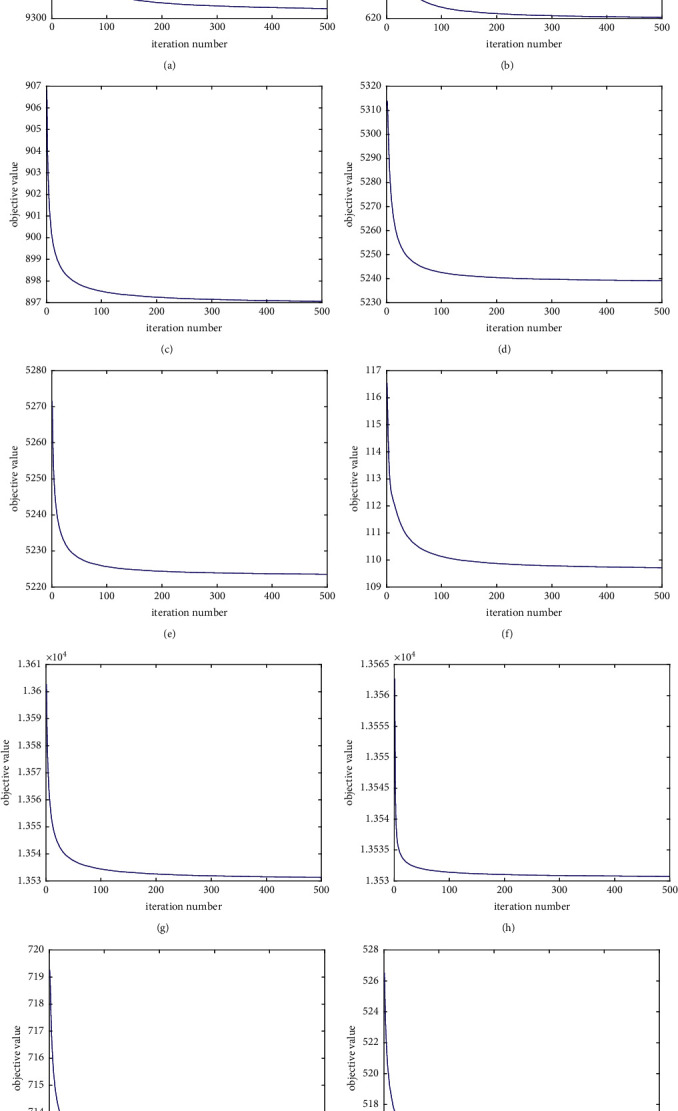
Convergence curves of the proposed GRDSR method on the ten datasets. (a) Annthyroid, (b) SpamBase, (c) WPBC, (d) Cardiotocography, (e) Ionosphere, (f) WBC, (g) Arrhythmia, (h) Pen digits, (i) Stamps, and (j) Heart.

The updating rules of GRDSR are essentially iterative and the convergence for the objective function value is theoretically guaranteed. Now, we investigate how fast the rules can reach convergence. We conduct the experiments at all datasets, and the results are shown on Figure 6. For each figure, *x*-axis is the iteration number and *y*-axis denotes the objective function value. It shows that the proposed GRDSR method can reach convergence after 100 iterations at most of the datasets.

### 4.7. Running Time

In order to show the efficiency of the proposed algorithm more intuitively, we test the running time of our proposed GRDSR method on each dataset. Our algorithm is implemented by MATLAB, and these experiments are carried out on PC machine with Intel i9 9900K 3.60 GHz and 32 GB memory. We record the running time when the iteration number is set to be 100 as report in Table 6. From the results, the running time of the proposed method is acceptable.

## 5. Conclusions

Different from the traditional MF-based methods, we propose a deep representation framework based on sparse representation named graph regularized deep sparse representation (GRDSR) to learn the deep feature representation for anomaly detection. In GRDSR, we first apply multilayers' factorization to extend the single matrix factorization. Next, we add the graph regularize term into each layer factorization to capture the intrinsic geometric structure information of the original data. Then, we introduce a sparisty constraint-based *l*_1_-norm to avoid the overfiting problem and extract more discriminative deep feature representations. Last, we utilize a criterion-based reconstruction error to detect anomaly data. The experiments are carried out on ten widely used datasets. According to the experimental results, we can learn that the proposed method outperforms the state-of-the-art approaches.

## Figures and Tables

**Algorithm 1:**
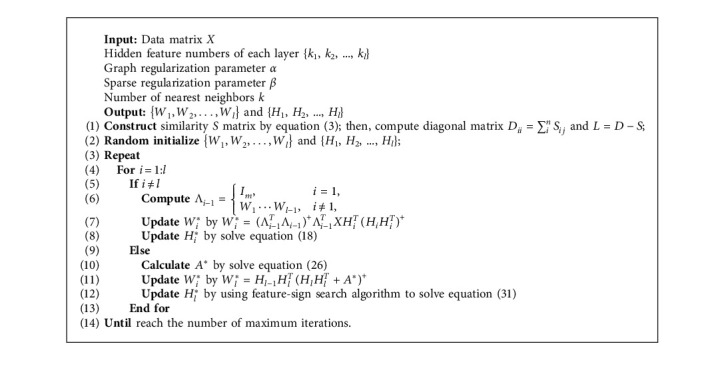
The optimization algorithm of GRDSC.

**Algorithm 2:**
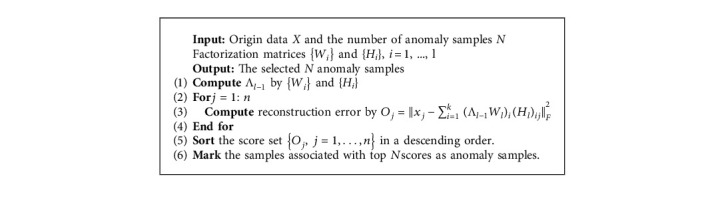
The procedure of anomaly detection

**Table 1:** Public benchmark datasets collected from real world.

Dataset	Number of sample	Feature dimension	Outliers (%)
Annthyroid	7200	21	347(4.82)
SpamBase	4601	57	280(6.09)
WPBC	198	33	47(23.73)
Cardiotocography	2126	21	86(4.05)
Ionosphere	351	32	126(35.90)
WBC	454	9	10(2.20)
Arrhythmia	450	259	12(2.67)
Pen digits	9868	16	20(0.20)
Stamps	340	9	16(4.71)
Heart	270	13	7(2.59)

**Table 2:** Settings for different methods.

Methods	Number of latent features	Other parameters
GraphSC	5	*k* = 5, 0-1 weighting (i.e., equation (3))
SR	5	
Offline NS-NMF	5	*α* = 0.8 and *γ* = 0.2
Online NS-NMF	5	*α* = 0.8 and *z* = 20
GNMF	5	*k* = 5, 0-1 weighting (i.e., equation (3))
SNMF	5	Heat kernel weighting (i.e., equation (4))
NMF	5	
Ours	[(*m*/2), (*m*/4)]	*k* = 5, 0-1 weighting (i.e., equation (3))

**Table 3:** Results of the NMF-based methods.

Dataset	GRDSR	GraphSC	SR	Offline NS-NMF	Online NS-NMF	NMF	GNMF	SNMF	Total anomalies
Annthyroid	**30**	17	16	27	15	15	13	10	347
SpamBase	77	61	61	47	41	32	36	23	280
WPBC	**14**	**14**	**14**	11	9	11	8	9	47
Cardiotocography	**32**	31	30	29	15	27	29	29	86
Ionosphere	108	**109**	108	92	74	79	74	33	126
WBC	**9**	8	8	**9**	7	2	8	8	10
Arrhythmia	**5**	4	3	4	3	2	3	3	12
Pen digits	**3**	3	0	0	1	0	0	0	20
Stamps	**4**	4	3	**4**	2	3	3	2	16
Heart	**5**	4	**5**	**5**	4	0	4	4	7

The bold values mean the best result for each dataset.

**Table 4:** Results of non-NMF-based methods.

Dataset	GRDSR	COF	LDF	kNN	ODIN	LOF	*k*NNW	SLOF	LoOP	INFLO	LDOF	FABOD	AER	DAGMM	Total anomalies
Annthyroid	30	5	5	3	3	3	3	3	3	3	3	3	10	**94**	347
SpamBase	**77**	55	48	76	65	55	76	44	55	55	52	64	31	61	280
WPBC	**14**	11	13	10	12	10	9	10	10	11	13	10	10	9	47
Cardiotocography	**32**	28	28	30	18	22	31	27	26	23	25	29	29	27	86
Ionosphere	**108**	107	104	107	99	105	**108**	104	101	101	101	**108**	63	79	126
WBC	**9**	8	8	8	4	8	8	6	4	4	4	7	6	2	10
Arrhythmia	**5**	**5**	**5**	3	3	3	3	3	3	3	3	3	2	2	12
Pen digits	3	3	1	0	0	1	1	1	1	0	1	1	0	**5**	20
Stamps	**4**	5	4	3	3	4	4	4	4	4	5	**6**	3	0	16
Heart	**5**	4	4	4	4	4	4	4	4	4	2	2	2	2	7

The bold values mean the best result for each dataset.

**Table 5:** The best settings for all datasets.

Datasets	*α*	*β*
Annthyroid	10^−2^	1
SpamBase	10^−3^	10^−3^
WPBC	10^−3^	1
Cardiotocography	10^−2^	10^−1^
Ionosphere	10^−2^	1
WBC	1	10^−3^
Arrhythmia	10^−2^	1
Pen digits	10^−3^	1
Stamps	10^−3^	10^−3^
Heart	10^−3^	10

**Table 6:** Running time on each of the dataset.

Datasets	Running time (s)
Annthyroid	163.32
SpamBase	61.35
WPBC	2.55
Cardiotocography	23.85
Ionosphere	4.33
WBC	2.34
Arrhythmia	15.21
Pen digits	232.84
Stamps	3.16
Heart	1.85

## Data Availability

The data used to support the findings of the study are derived from public domain resources.
